# Chemical profiling, bioactive compounds, antioxidant, and anti-inflammatory activities of Indonesian propolis extract produced by *Tetragonulalaeviceps*

**DOI:** 10.1016/j.heliyon.2024.e38736

**Published:** 2024-09-30

**Authors:** Muhammad Yusuf Abduh, Tri Ramadianti Shafitri, Elfahmi Elfahmi

**Affiliations:** a School of Life Science and Technology, Institut Teknologi Bandung, Indonesia; b University Center of Excellence for Nutraceutical, Bioscience and Biotechnology Research Center, Institut Teknologi Bandung, Indonesia; c School of Pharmacy, Institut Teknologi Bandung, Indonesia

**Keywords:** Antioxidant activity, Anti-inflammatory activity, Bioactivity, Chemical profile, Propolis

## Abstract

Propolis produced by stingless bees contains various chemical compounds that contribute to its bioactivity. The availability of certain plants at the growth site and the propolis's geographic origin have a significant impact on its chemical composition. The objective of this study was to examine the chemical profile, yield, total flavonoid and phenolic content, and *in vitro* antioxidant and anti-inflammatory effects of propolis extract from 10 distinct locations in Indonesia. The yield of propolis extract investigated in this study lies in the range of 26.25 ± 1.76 to 43.25 ± 3.60 %. The total phenolic content of the propolis extract varies from 50.03 ± 3.40–98.03 ± 13.94 mg GAE/g whereas the total flavonoid content of the propolis extract varies from and 0.70 ± 0.08–57.76 ± 0.67 mg QE/g. The antioxidant activity of the propolis extract in terms of IC_50_ values lies in the range of 332.07 ± 6.12 to 831.48 ± 29.48 ppm whereas the anti-inflammatory activity of the propolis extract in terms of IC_50_ values lies in the range of 28.69 ± 4.95 to 44.12 ± 19.22 ppm. Both the total flavonoid and phenolic content of the propolis extract from various locations were correlated with the antioxidant activities and anti-inflammatory activities. The results indicate that there was a significant negative correlation between the total flavonoid and phenolic content of the propolis extract with the antioxidant activity. However, the anti-inflammatory activity was not strongly correlated with the total flavonoid and phenolic content of the propolis extract. There were 36 volatile compounds in the propolis extract as identified by the Gas Chromatography–Mass Spectrometry with triterpenoid as the major substances (28.66–44.86 %). The presence of anti-inflammatory compounds particularly α, β-Amyrin (2.2–6.52 %) and lupeol (2–4.72 %) in the propolis extract highlights the potential of propolis in health and medicine application.

## 1 Introduction

Bees produce propolis, a sticky substance, by combining resinous material from plant sources with their saliva. They then use this substance to build their hives and defend themselves against predators and pathogens [1,2]. Numerous studies have reported that propolis exhibits potential antioxidant, antibacterial and anticancer activity [[3], [4], [5]]. The bioactivity of propolis is highly dependent on its chemical composition which is influenced by various factors such as geographic regions, climate, plant sources and bee species [6,7] especially the stingless bees.

The stingless bee belongs to the tribe Meliponini comprises of 32 genera and more than 500 species [6]. According to Kothai and Jayanthi, stingless bees produce more propolis (up to 6-times) than the propolis produced by honeybees [8]. In terms of quality, Muhammad and Sarbon reported that propolis produced by stingless has a higher antioxidant activity and phenolic content as compared to propolis produced by stingless bees [9]. In another study, Abduh et al. discovered that propolis produced by *Tetragonula laeviceps* has a higher phenolic and flavonoid content than propolis produced by *Tetragonula drescheri*, and *Tetragonula biroi* [7].

Systematic studies that investigate the correlation between chemical composition and bioactivity of propolis produced by stingless bees from different geographical regions are very scarce. Several studies have been conducted to investigate the chemical profile and bioactivity of propolis extract produced by honeybees. For instance, Bittencourt et al. used Gas Chromatography–Mass Spectrometry to determine the chemical profile of brown and green propolis extract from Brazil whereas Falcão et al. used Liquid-chromatography–Mass Spectrometry to determine the phenolic profile of 40 propolis extract from Portugal [10,11]. The findings demonstrated that the composition of the propolis extract differs from one source to another. As such highlights the importance of investigating further the composition of propolis extracts obtained from different geographical origin to identify potential bioactive compounds in propolis extracts.

At present, there are not yet systematic studies that report the yield of propolis extract together with the correlation of chemical profile with bioactivity of propolis extract produced by *T. laeviceps*. Hence, this study aimed to determine the propolis extract yield and chemical composition of propolis extract produced by *T. laeviceps* from different locations in Indonesia as well as their phenolic and flavonoid content, antioxidant, and anti-inflammatory activities. In addition, multivariate statistical analysis was applied in this study to determine the correlation between chemical composition and bioactivity of the propolis extract.

## 2 Materials and methods

### 2.1 Materials

**Fig. 1:**
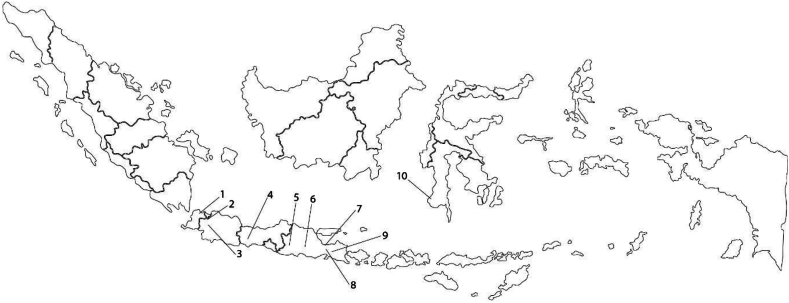
Geographical locations of crude propolis origin used in this study. Note: (1) Serang, Banten, (2) Depok, West Java, (3) Bogor, West Java, (4) Banyumas, Central Java, (5) Ponorogo, East Java, (6) Kediri, East Java, (7) Probolinggo, East Java, (8) Lumajang, East Java, (9) Jember, East Java, and (10) Gowa, South Sulawesi.

Crude propolis used in this study was obtained from stingless bee (*T. laeviceps*) farmers from various locations in Indonesia as shown in Fig. 1. Upon receipt, the crude propolis was stored at −20 °C until further processing. Ethanol, Methanol, Folin-Ciocalteu, sodium carbonate and other reagents were of analytical grade obtained from Merck Co. (Darmstadt, Germany). Quercetin, gallic acid, and other reagents were of analytical grade obtained from Sigma-Aldrich Chemical Co. (St. Louis, USA).

### 2.2 Extraction of bioactive compounds from crude propolis

Extraction of bioactive compounds was carried out using methods as suggested by Abduh et al. [7] with slight modifications. Approximately 10 g of crude propolis was extracted using 750 mL of ethanol in the Soxhlet apparatus operated at 85 °C for 2 h. After that, the extract was filtered using Whatman filter paper No. 2 and the solvent was evaporated using a rotary vacuum evaporator at 55 °C until all the solvent had fully evaporated and then weighed before stored at −20 °C until further processing. The yield of propolis extract was determined using equation (1).

(1) $$Yield\left(\%\right)=\left(\frac{m_{propolis\;extract}\;\left(g\right)}{m_{crude\;propolis}\;\left(g\right)}\right)\times 100\%$$

### 2.3 Determination of total phenolic content in propolis extract

A standard curve was prepared by mixing the propolis extract (1000 ppm) with gallic acid (0–100 ppm) and then dissolved in methanol as suggested by Abduh et al. [7]. To determine the total phenolic content of the propolis extract, approximately 0.5 mL of the propolis extract sample was mixed with 2.5 mL of 10 % (vol/vol) Folin-Ciocalteu followed with the addition of 2 mL of 7.5 % (weight/volume) sodium carbonate solution before incubation at room temperature (27 °C) for 30 min. Absorbance of the sample was measured using a UV–Vis spectrophotometer at a wavelength of 765 nm. The total phenolic content of the propolis extract was considered as the equivalent concentration of gallic acid in the sample. The linear regression equation for gallic acid concentration and absorbance were determined to obtain the concentration gallic acid in the propolis extract (mg GAE/mL). The total phenolic content (mg GAE/g) of the propolis extract was determined using equation (2).

(2) $$Total\;phenolic\;content\;\left(mg\;GAE/g\right)=\frac{Total\;phenolic\;content\;\left(mg\frac{GAE}{mL}\right)\times V_{sample}\left(mL\right)}{m_{propolis\;extract}\;\left(g\right)}$$

### 2.4 Determination of total flavonoid content in propolis extract

A standard curve was prepared by mixing the propolis extract (1000 ppm) with quercetin (0–100 ppm) and then dissolved in methanol as suggested by Abduh et al. [7]. To determine the total flavonoid content of the propolis extract, approximately 2 mL of the propolis extract sample was mixed with 2 mL of 2 % AlCl_3_ solution. The mixture was homogenized using a vortex and then incubated in the dark for 30 min. Absorbance of the sample was measured using a spectrophotometer UV–Vis spectrophotometer at a wavelength of 415 nm. The total flavonoid content of the propolis extract was determined as the equivalent concentration of quercetin in the propolis extract. The linear regression equation for concentration of quercetin and absorbance were determined to obtain the concentration quercetin in the propolis extract (mg QE/mL). The total flavonoid content (mg QE/g) of the propolis extract was determined using equation (3).

(3) $$Total\;flavonoid\;content\;\left(mg\;QE/g\right)=\frac{Total\;flavonoid\;content\;\left(mg\frac{QE}{mL}\right)\times V_{sample\;\left(mL\right)}}{m_{propolis\;extract}\left(g\right)}$$

### 2.5 Determination of antioxidant activity of propolis extract

Antioxidant activity of the propolis extract was determined using 2,2-diphenyl-1-picrylhydrazyl (DPPH). The propolis extract was dissolved in methanol to prepare a sample solution with a concentration range of 0–1000 ppm with methanol as a blank. The sample solution (1 mL) was mixed and homogenized with 4 mL of 0.05 mM DPPH solution using a vortex and then incubated in the dark for 30 min [12]. Absorbance of the sample was measured using a UV–Vis spectrophotometer at a wavelength of 514 nm. The antioxidant activity of the propolis extract as indicated by the DPPH activity was calculated using equation (4).

(4) $$Antioxidant\;activity\;\left(\%\right)=DPPH\;scavenging\;activity=\frac{\left(A_{blank}-A_{sample}\right)}{A_{blank}}\times 100$$

The concentration of sample required to inhibit DPPH by 50 % (IC_50_) was determined by plotting the sample concentration as x and the percentage of DPPH scavenging activity as y to obtain the linear regression equation. A smaller value of IC_50_ corresponds to a higher antioxidant activity [12].

### 2.6 Determination of anti-inflammatory activity of propolis extract

The anti-inflammatory activity of the propolis extract was determined by calculating the ability of the propolis extract to inhibit denaturation of the bovine serum albumin (BSA) protein following the method described in Rahman et al. [13]. Initially, a solution of phosphate buffered saline (PBS) was prepared by dissolving 8 g NaCl, 0.2 g KCl, 1.44 g ${\text{Na}}_{2}{\text{HPO}}_{4}$, 0.24 g ${\text{KH}}_{2}{\text{PO}}_{4}$ in 800 mL of distilled water. Then, the pH of the PBS solution was adjusted to pH 6.3 using 1N HCl and distilled water was added until the volume of the PBS solution became 1 L. After that, a BSA solution was prepared by dissolving 500 mg of BSA in 100 mL of distilled water. The propolis extract was dissolved in methanol to prepare a sample solution with a concentration range of 0–1000 ppm with methanol as a blank. After that, 2 mL of propolis extract was mixed with 0.2 mL of BSA (0.5 %) and incubated in a water bath at 37 °C for 15 min followed by heating at 70 °C for 5 min and then allowed to cool down at room temperature (27 °C). Then, the mixture was mixed with 2.8 mL of PBS and homogenized using a vortex. The absorbance of the sample was measured using a UV–Vis spectrophotometer at a wavelength of 660 nm. The inhibition percentage of protein denaturation was calculated using equation (5).

(5) $$\%\;inhibition\;denaturation\;of\;BSA=\frac{\left({A_{sample}-A}_{blank}\right)}{A_{blank}}\times 100\%$$

The concentration of sample required to inhibit protein denaturation by 50 % (IC_50_) was determined by plotting the sample concentration as x and the percentage of protein denaturation inhibition as y to obtain the linear regression equation with a smaller value of IC_50_ corresponds to a higher anti-inflammatory activity. The anti-inflammatory activity of the propolis extract was compared to the positive control in the form of diclofenac potassium (a commercial anti-inflammatory drug) [13,[34], [35], [36], [37]].

### 2.7 Untargeted chemical profiling of propolis extract

Untargeted chemical profiling of volatile compounds in the propolis extract was carried out using a Gas Chromatography – Mass Spectrometry (GS-MS) with ethanol as the standard compound as suggested by Bittencourt et al. [10]. The propolis extract samples were re-dissolved in analytical grade ethanol with a concentration of 10,000 ppm for the GC-MS analysis. The analysis was performed with Agilent 7890A coupled to Agilent 5977B GC/MS MS detector, using DB-5MS (5%-phenyl-methylpolysiloxane; 30 m × 0.25 mm), using helium gas as a carrier gas at a flow rate of 1 mL/min as described by Bittencourt et al. [10]. The oven temperature was initially held at 80 °C and increased to 180 °C at the rate of 7 °C/min and held for 1 min. Finally, the temperature was increased to 320 °C at a rate of 7 °C/min, the split ratio was 5:1 with an auxiliary temperature of 250 °C. The chemical compounds contained in the propolis extract were identified based on their retention time by comparison of their mass spectra with data from National Institute of Standard and Technology (NIST) library.

### 2.8 Data analysis

The results obtained in this study were analysed using *t*-test (*P* < 0.05), Principal Component Analysis (PCA), and heatmap correlation using MetaboAnalyst version 5.0.

## 3 Results and discussion

### 3.1 Yield of propolis extract obtained from 10 different sample locations in Indonesia

**Fig. 2:**
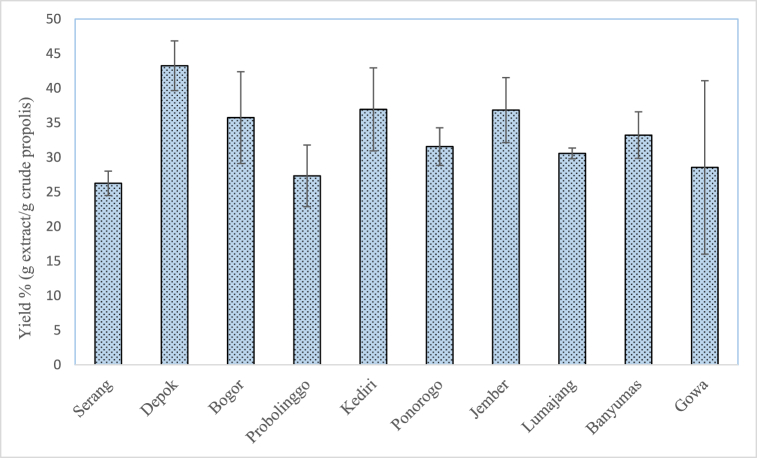
Yield of propolis extract from different geographical origin.

Fig. 2 shows the yield of propolis extract obtained in this study which lies in the range of 26.25 ± 1.76 to 43.25 ± 3.60 %. These values are higher than the yield of Indian propolis (3.95–12.75 %), propolis from Banten and South Sulawesi, Indonesia (4.91–12.81 %) as reported in previous studies, respectively (Table 1) [14,15]. These differences may be due to different extraction conditions methods particularly the type and concentration of extracting solvent, temperature and propolis to solvent ratio. In the study by Ramnath & Venkataramegowda, the crude propolis was extracted using 70 % ethanol with intermittent shaking overnight at room temperature (25 °C) [14]. Whereas in the study by Fikri et al., the crude propolis was extracted by ultrasound-assisted extraction using 75 % ethanol with a ratio of 1:10 [15]. In this study, crude propolis was extracted using a Soxhlet extraction method at a higher ethanol concentration (≥99.5 %), crude propolis to solvent ratio (1:75), and temperature (85 °C) thereby resulting higher extract yield as suggested by previous studies [15,16]. The results obtained in this study are in line with the previous study by Abduh et al. that the greatest propolis extract yield (32.45 %) was obtained when the crude propolis was extracted using a Soxhlet extraction method as compared to the reflux and maceration methods with ethanol (80 %) as the extracting solvent [7].

**Table 1:** Yield, bioactive compound, and bioactivity of propolis from various locations in Indonesia and other countries.

Source of crude propolis	Yield % (g extract/g crude propolis)	Total Phenolic Content (mg GAE/g)	Total Flavonoid Content (mg QE/g)	Antioxidant Activity (IC_50_, ppm)	Anti-inflammatory Activity (IC_50_, ppm)	Source
Serang, Banten, Indonesia	26.2 5 ± 1.76^a^	60.81 ± 2.98^b^	3.13 ± 0.57^a^	694.47 ± 15.60^e^	32.70 ± 0.30^a,b,c^	This study
Depok, West Java, Indonesia	43.25 ± 3.60^b^	80.60 ± 3.20^d^	18.24 ± 0.49^c^	404.35 ± 15.57^b^	33.08 ± 1.52^a,b,c^	This study
Bogor, West Java, Indonesia	35.747 ± 6.63^a,b^	93.46 ± 10.43^d^	57.76 ± 0.67^f^	396.47 ± 35.59^b^	42.05 ± 4.90^b,c^	This study
Probolinggo, Central Java, Indonesia	27.33 ± 4.46^a^	73.43 ± 3.54^c^	3.89 ± 0.45^a^	560.42 ± 17.40^d^	28.69 ± 4.95^a^	This study
Kediri, East Java, Indonesia	36.94 ± 6.00^a,b^	50.03 ± 3.40^a^	0.70 ± 0.08^a^	831.48 ± 29.48^f^	31.60 ± 13.96^a,b^	This study
Ponorogo, East Java, Indonesia	31.56 ± 2.72^a^	63.01 ± 4.61^b^	24.30 ± 1.05^d^	557.51 ± 28.04^d^	35.17 ± 1.83^a,b,c^	This study
Jember, East Java, Indonesia	36.83 ± 4.71^a,b^	86.60 ± 11.82^d^	19.23 ± 3.91^c^	482.84 ± 9.91^c^	42.99 ± 11.65^b,c^	This study
Lumajang, East Java, Indonesia	30.57 ± 0.78^a^	98.03 ± 13.94^d^	20.41 ± 2.02^d^	367.93 ± 24.60^a,b^	32.42 ± 1.83^a,b,c^	This study
Banyumas, East Java, Indonesia	33.217 ± 3.37^a,b^	61.91 ± 2.97^b^	14.12 ± 2.54^b^	491.97 ± 16.08^d^	44.12 ± 19.22^c^	This study
Gowa, South Sulawesi, Indonesia	28.55 ± 12.54^a^	94.17 ± 9.82^d^	43.86 ± 3.75^e^	332.07 ± 6.12^a^	36.00 ± 5.49^a,b,c^	This study
Jatinangor, West Java, Indonesia	13.38–32.45	98.13–343.93	4.41–35.77	–	–	[7]
Jatinangor, West Java, Indonesia	–	32.23–112.13	1.78–3.02	–	–	[5]
Cileunyi, West Java, Indonesia	–	–	11.4 ± 4.4	–	–	[2]
Cibodas, West Java, Indonesia	–	–	14.8 ± 6.2	–	–	[2]
Banten, Indonesia	4.91–7.72	15.52–15.64	0.76–1.80	503.93–568.99	–	[15]
South Sulawesi, Indonesia	10.97–12.85	15.32–22.30	1.50–3.39	543.09–810.75	–	[15]
South Kalimantan, Indonesia	9.16–11.78	10.30–28.65	1.42–2.61	452.52–1027.29	–	[15]
South Sulawesi Indonesia	–	98.08 ± 0.05	15.89 ± 0.92	–	–	[17]
Malaysia	–	7.60–13.21	34.17–34.53	3950–6140	–	[16]
Mexico	–	6.9–49.3	73–379	–	–	[18]
Mexico	–	18.65–226.14	6.51–48.54	–	–	[19]
Chili	–	14.6–36.4	2.1 14.8	–	–	[20]
Morocco	–	141.4 6 ± 1.67	98.33 ± 1.19	20 ± 2	125 - 250∗	[21]
India	3.95–12.75	8.25–15.56	–	333,48–600,88	–	[14]
Brazil	–	100.7 ± 6.47	–	913.18 ± 170.96	–	[22]
Venezuela	–	19.1–182	<1–60.2	39–221	–	[23]
Lithuania	–	–	–	49.92–171.29	–	[24]

Note: Different lowercase letters on the same line indicate that there is a significant difference (P-value <0.05). ∗Concentration of propolis (μg/mL) used to examine anti-inflammatory cytokines production.

### 3.2 Untargeted chemical profiling of volatile compound in propolis extract

**Fig. 3:**
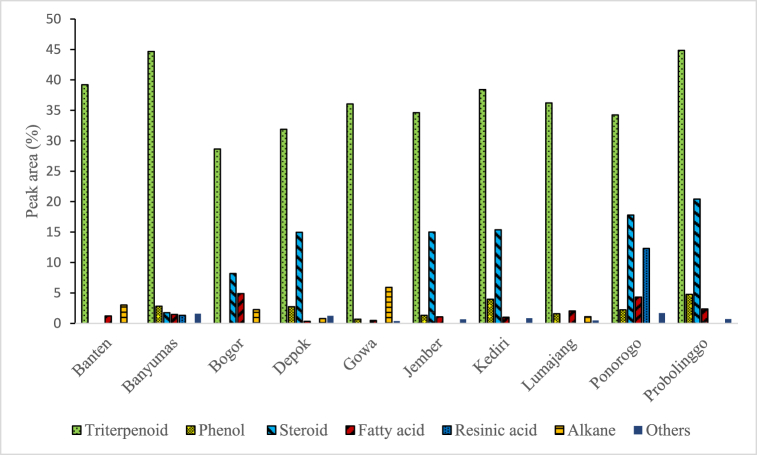
Major volatile compounds in propolis extract from different geographical origin.as detected by GC-MS.

In this study, 36 volatile compounds in the propolis extract from different locations in Indonesia had been determined using GC-MS and the results are shown in Table 2. In general, the detected compounds can be classified into 7 major categories with triterpenoid that has multifunctional bioactivities as the major substances (28.66–44.86 %) as shown in Table 2 and Fig. 3. Similarly, a total of 35 different chemical substances were identified in Malaysian stingless bee propolis [16]. However, the propolis contains less amount of terpenoid (14.3 %) with reducing sugars (31.4 %) as the major compounds due to the used of water as the extraction solvent. According to Fikri et al., extraction of propolis with water typically produces more carbohydrates, terpenes, and other nonphenolic compounds while extraction of propolis with ethanol produces more terpenoid compounds as obtained in this study [15].

**Table 2:** Volatile compounds in propolis extract from different geographical origin.as detected by GC-MS.

No	Groups	Compounds	Peak Area (%)									
			Banten	Banyumas	Bogor	Depok	Gowa	Jember	Kediri	Lumajang	Ponorogo	Probolinggo
1	Triterpenoid	Cycloartenol acetate	23.78	24.04	18.49	25.26	19.94	24.79	24.60	25.62	14.84	22.30
2		α-Amyrin	–	8.15	–	–	–	–	7.43	–	7.00	8.88
3		β-Amyrin	2.23	3.56	1.67	1.97	6.52	1.44	2.20	3.37	4.07	6.25
4		Lanosterol	10.01	5.60	–	2.66	5.03	5.10	1.79	4.89	3.62	5.13
5		Lupeol	2.37	3.32	3.05	2.00	3.18	2.77	2.40	2.34	4.72	2.30
6		Obtusifoliol	–	–	5.02	–	–	–	–	–	–	–
7		Squalene	0.82	–	0.43	–	–	0.52	–	–	–	–
8		Supraene	–	–	–	–	1.37	–	–	–	–	–
9	Phenol	Cardanol	–	0.63	–	0.68	–	0.57	1.09	0.80	–	1.05
10		Cardanol monoene	–	1.40	–	–	–	–	0.61	–	1.36	1.26
11		3-Heptadecylphenol	–	0.50	–	0.81	0.39	0.46	1.47	0.47	0.88	1.60
12		Hydroginkol	–	0.30	–	1.26	0.30	0.30	0.79	0.32	–	0.87
13	Steroid	Lupen-3-one	–	1.76	8.19	14.97	–	15.01	15.39	–	17.80	20.44
14	Fatty acid	cis-Vaccenic acid	1.22	0.89	–	–	–	0.78	0.60	1.07	2.67	1.67
15		Ethyl Oleate	–	0.58	–	0.33	0.28	0.29	0.41	0.68	0.77	0.36
16		Hexadecanoic acid, ethyl ester	–	–	3.02	–	–	–	–	–	–	–
17		Hexadecanoic acid, methyl ester	–	–	–	–	–	–	–	–	0.37	0.35
18		Linoleic acid ethyl ester	–	–	1.04	–	–	–	–	0.30	–	–
19		n-Hexadecanoic acid	–	–	0.84	–	0.21	–	–	–	0.52	–
20	Resinic acid	Abietic acid	–	0.31	–	–	–	–	–	–	2.62	–
21		Dehydroabietic acid	–	0.47	–	–	–	–	–	–	5.21	–
22		Isopimaric acid	–	0.54	–	–	–	–	–	–	4.48	–
23	Alkane	Heneicosane	–	–	0.35	–	–	–	–	–	–	–
24		Hentriacontane	–	–	–	–	2.93	–	–	–	–	–
25		Heptacosane	1.22	–	1.61	0.79	–	–	–	1.11	–	–
26		Hexacosane	1.80	–	–	–	–	–	–	–	–	–
27		Octacosane	–	–	–	–	3.00	–	–	–	–	–
28		Tricosane	–	–	0.30	–	–	–	–	–	–	–
29	Others	Hydroxydehydrostevic acid	–	–	–	–	–	–	–	–	0.27	–
30		Behenyl chloride	–	–	–	–	–	0.67	–	–	–	–
31		Bis(2-ethylhexyl) phthalate	–	–	–	–	–	–	0.53	–	0.86	–
32		Diethyl Phthalate	–	–	–	–	–	–	0.33	–	0.57	–
33		Ethanol, 2,2′-oxybis-	–	1.61	–	–	–	–	–	0.49	–	–
34		Ginsenol	–	–	–	0.50	–	–	–	–	–	0.72
35		Cedrol	–	–	–	–	0.39	–	–	–	–	–
36		Copaene	–	–	–	0.75	–	–	–	–	–	–

From Table 2, it can be observed that not all compounds were detected in all the samples investigated in this study. The most abundant compound found in all propolis samples was cycloartenol acetate with a peak area of 14.84–25.62 % (Table 2). This compound is known to have an anti-mosquito larvicidal activity [25]. Besides that, lupeol compound was also found in all samples while lanosterol was found in all samples except Bogor propolis. Lupeol is a metabolite compound that has pharmacological activities such as anticancer, antioxidant, anti-inflammatory, and antimicrobial activities while lanosterol is a tetracyclic triterpenoid which has a role in reducing cataracts [26,27]. Similar findings were also reported by Bittencourt et al. [10] where ethyl oleate, hexadecanoic acid, and lupeol were also found in Brazilian propolis.

β-Amyrin was also found in all samples while α amyrin was only found in Banyumas and Kediri propolis. According to Melo et al., α, β-amyrin is a pentacyclic triterpene and pharmacological studies have revealed its anti-inflammatory activity and has the potential to combat acute pancreatitis [28]. In this study, unique compounds were identified, particularly ginsenol and copaene, which were only found in Depok propolis. Ginsenol is a sesquiterpenoid alcohol that has antifungal properties [29]. Supraene or squalene was also found in Banten, Bogor, Gowa, and Jember propolis. Squalene is a natural triterpene compound that is found in abundance in nature from the sterol biosynthetic pathway [30].

**Fig. 4:**
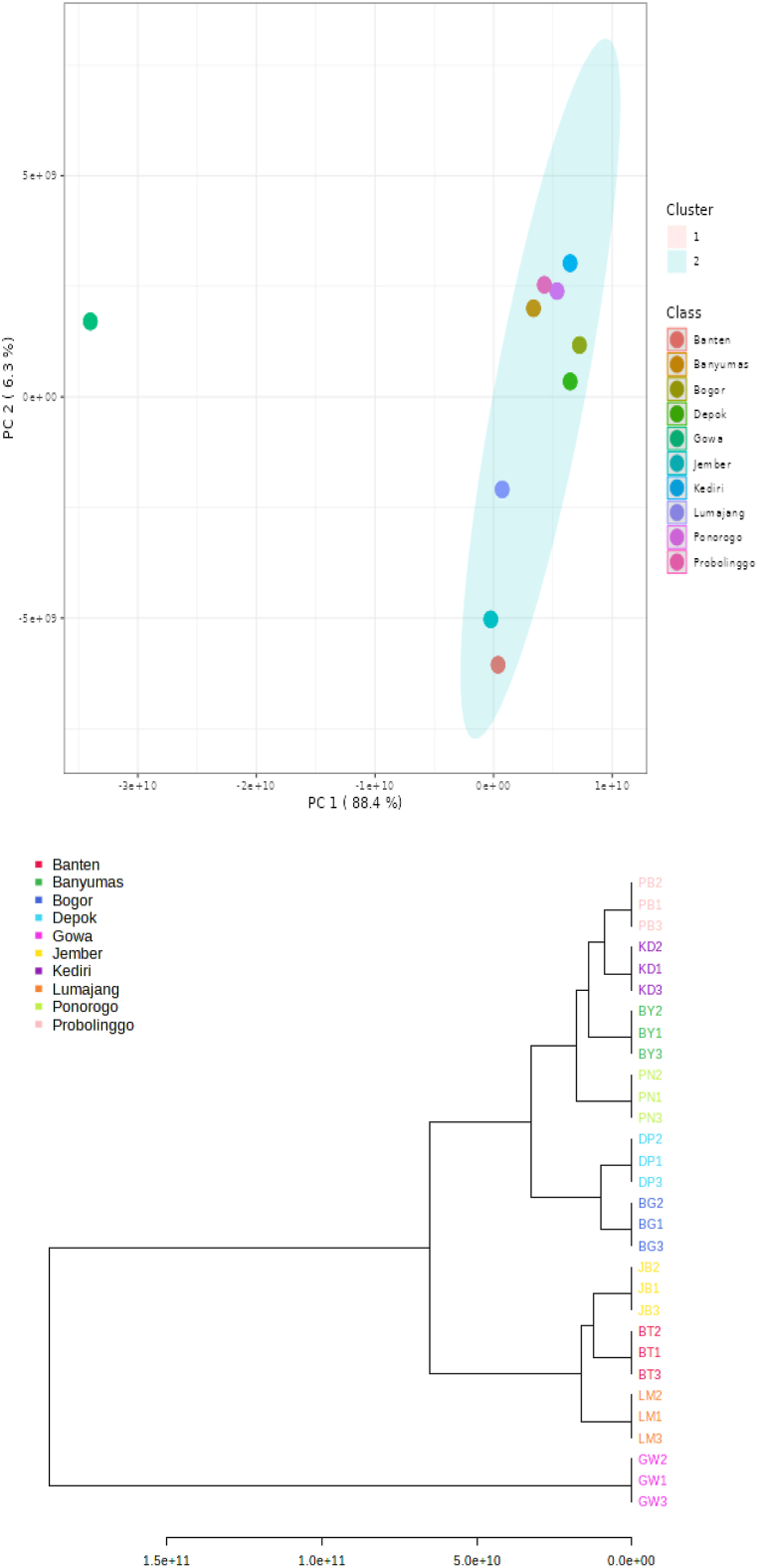
Principal component analysis (top) and hierarchical cluster analysis using the Ward method of chemical compounds detected in 10 propolis from different locations in Indonesia. (BT) Serang, Banten, (DP) Depok, West Java, (BG) Bogor, West Java, (BY) Banyumas, Central Java, (PN) Ponorogo, East Java, (KD) Kediri, East Java, (PB) Probolinggo, East Java, (LM) Lumajang, East Java, (JB) Jember, East Java, and (GW) Gowa, South Sulawesi (bottom).

**Fig. 5:**
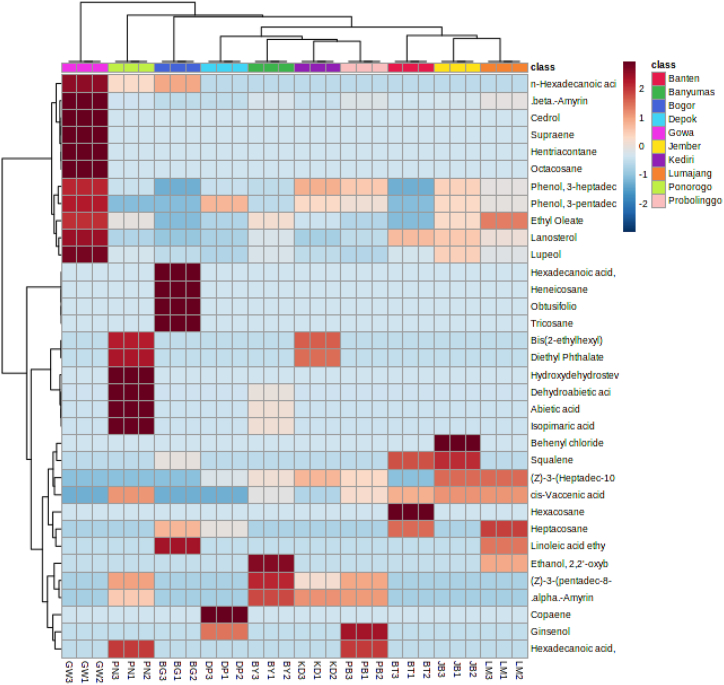
Heatmap representation of chemical compounds detected in 10 propolis from different locations in Indonesia. (BT) Serang, Banten, (DP) Depok, West Java, (BG) Bogor, West Java, (BY) Banyumas, Central Java, (PN) Ponorogo, East Java, (KD) Kediri, East Java, (PB) Probolinggo, East Java, (LM) Lumajang, East Java, (JB) Jember, East Java, and (GW) Gowa, South Sulawesi.

In this study, a principal component analysis (PCA) was carried out to explain the variance in the data set and differentiate one propolis sample based on chemical profile as detected by the GC-MS and the results are shown in Fig. 4, Fig. 5. Principal component 1 explains 88.4 % of the total variance while principal component 2 explains 6.3 % of the total variance. PCA plot analysis clearly differentiates Gowa propolis from other propolis samples. This is very interesting to be further investigated because Gowa propolis is the only propolis taken from Sulawesi Island while other propolis samples were collected from different cities in the Java Island. Putting the other compounds aside, there were cedrol and octacosane compounds detected only in Gowa propolis. Cedrol is an essential oil component found in Juniperus species and has immunomodulatory activity whereas octasonsane is a plant metabolite that has moderate antioxidant activity and can improve wound healing [31,32]. The different chemical profiles in all the samples investigated in this study content may be due to different botanical sources around the cultivation site of the *T. laeviceps* [16].

The phytochemical composition of propolis exhibits a direct correlation with the botanical sources surrounding the beehive, serving as source of resinous materials [38]. Plant species such as *Populus* spp., *Pinus* spp., *Prunus* spp., *Acacia* spp., *Betula pendula*, *Aesculus hippocastanum*, and *Salix alba* represent the primary resin sources in temperate zones, owing to their widespread distribution across temperate regions of Europe, Asia, and North America [39]. Polish and Romanian propolis are categorized as poplar-type propolis, while Lithuanian propolis encompasses both balsamic and black poplar types, characterized by a predominance of p-coumaric acid and black poplar-caffeic acid, respectively [40]. Additional well-established propolis types include green Brazilian propolis, predominantly derived from *Baccharis dracunculifolia*, red Brazilian propolis sourced from *Dalbergia ecastophyllum* and *Symphonia globulifera*, European propolis obtained from *Populus nigra* L., Russian propolis derived from *Betula verrucosa* Ehrh, and Cuban and Venezuelan red propolis sourced from *Clusia* spp [38,40]. Turkish propolis investigations have revealed a preference for plants belonging to the Asteraceae, Boraginaceae, Brassicaceae, Fabaceae, and Salicaceae families [41].

Different plant species contribute unique phytoconstituents to the propolis matrix, thereby influencing its biological properties. This diversity stems from the bees' selective resin collection behavior, guided by factors such as proximity, resource availability, and potential plant toxicity [39]. This selectivity leads to variations in the chemical composition of propolis. Bioactive compounds within propolis, such as flavonoids like pinocembrin and pinostrobin, play a crucial role in their antioxidant and anti-inflammatory properties [39]. These compounds can be traced back to the specific botanical tapestry surrounding the beehive. However, in this study there was no analysis of possible botanical sources and their influence on geographical origin and the chemical characterization of propolis. Future studies need to be carried out for further investigation.

Based on the hierarchical cluster analysis of chemical compounds detected in the propolis extract using the Ward method, there were 2 large clusters: cluster 1 was Gowa propolis, and cluster 2 comprised of the remaining samples particularly Banten, Banyumas, Bogor, Depok, Jember, Lumajang, Kediri, Ponorogo and Probolinggo propolis (Fig. 2, Fig. 3). In cluster 2, there were 2 subclusters, the first subcluster comprised of Lumajang, Banten and Jember propolis whereas the second subcluster comprised of Ponorogo, Kediri, Banyumas, Probolinggo, Depok and Bogor propolis.

### 3.3 Total phenolic content of propolis extract

**Fig. 6:**
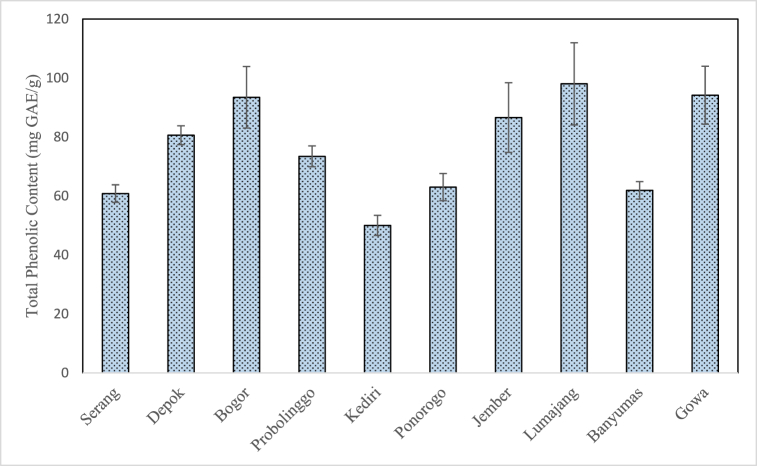
Total phenolic content of propolis extract from different geographical origin.

Fig. 6 shows the total phenolic content of propolis extract obtained in this study which lies in the range of 50.03 ± 3.40 to 98.03 ± 13.94 mg GAE/g. These values are still within the broad range as reported in previous studies as shown in Table 1 (8.25–226.14 mg GAE/g). The differences in the reported total phenolic content may be due to several factors such as stingless bee species, botanical sources, extraction, and solvent methods [16] as well as the methods used to determine the total phenolic content. For instance, Touzani et al. used a Reverse-Phase High Performance Liquid Chromatography to determine the total phenolic content whereas this study and several other studies used a Folin-Ciocalteau method [14,[21], [22], [23], [24]].

The highest total phenolic content in this study was observed in Lumajang propolis (98.03 ± 13.94 mg GAE/g) whereas the lowest total phenolic content in this study was observed in Kediri propolis (50.03 ± 3.40 mg GAE/g). In this study, the total phenolic content was set equivalent to gallic acid which means that the content of other types of phenolic compounds were not determined. Diverse results were also found in the phenolic content, where gallic acid (0.16–0.38 mg/g) was relatively smaller than p-Coumaric acid (37.54–116.95 mg/g) and other phenolic standards including caffeic acid, ferulic acid, hydroxybenzoic acid, and gentisic acid.

### 3.4 Total flavonoid content of propolis extract

**Fig. 7:**
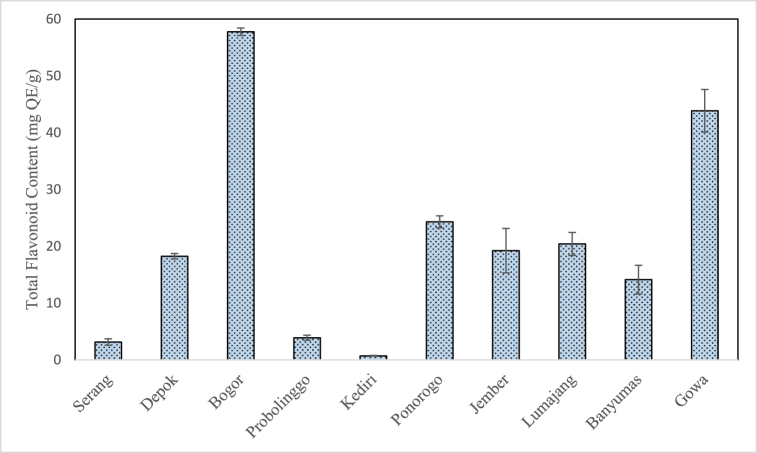
Total flavonoid content of propolis extract from different geographical origin.

Fig. 7 shows the total flavonoid content of propolis extract obtained in this study which lies in the range of 0.70 ± 0.08 to 57.76 ± 0.67 mg QE/g. These values are still within the broad range as reported in previous studies as shown in Table 1 (0.7–98.33 mg QE/g). The highest total flavonoid content in this study was observed in Bogor propolis (57.76 ± 0.67 mg QE/g) whereas the lowest total flavonoid content in this study was observed in Kediri propolis (0.7 ± 0.08 mg QE/g).

Previous research found that the flavonoid content of Cibodas and Cileunyi (Indonesia) propolis varies between 11.4 ± 4.4 and 14.8 ± 6.2 mg QE/g respectively [2]. This value resembles the flavonoid content of Depok propolis (18.24 ± 0.49 mg QE/g) and Banyumas (14.12 ± 2.54 mg QE/g) propolis. The total flavonoid content of Kediri propolis closely resembles the values reported in Banten (Indonesia) propolis (0.76–1.80 mg QE/g) as reported by Fikri et al. [15]. In contrast, another research found that Bogor propolis contains flavonoids of 2.9 mg QE/g [22]. Meanwhile, in this study Bogor propolis had the highest flavonoid content compared to other propolis (57.76 ± 0.67 mg QE/g). According to Salleh et al., differences in the reported total flavonoid content may be due to several factors such as stingless bee species, botanical sources as well as extraction and solvent methods [16].

In addition, the reported total flavonoid content also depends on the determination method. The total flavonoid content of propolis extract reported by de Fransisco et al. and Mohtar et al. was determined by a colorimetric assay based on the formation of flavonoid-aluminum [22,23]. In this study, the total flavonoid content was considered equivalent to quercetin which means that the concentration of other types of flavonoid compounds were not determined. Socha et al. investigated the flavonoid content of propolis extract with different standards and found that the quercetin content was relatively lower (4.13–8.06 mg/g) while the galangin content was relatively higher (12.65–35.08 mg/g) compared to other flavonoid standards including chrisin, hesperetin, kaempferol, and naringenin [33]. As such highlights that the reported total flavonoid content highly depends on the type of assay and standards used in the determination method.

### 3.5 Antioxidant activity of propolis extract

**Fig. 8:**
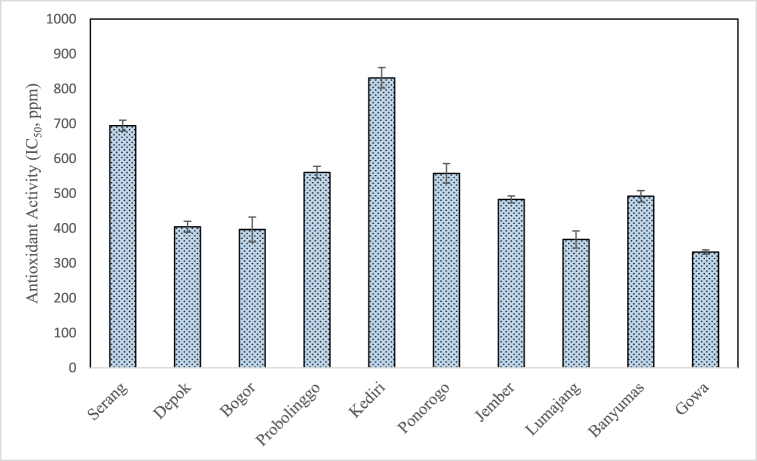
Antioxidant activity of propolis extract from different geographical origin.

Fig. 8 shows the antioxidant activity of propolis extract obtained in this study which lies in the range of 332.07 ± 6.12 to 831.48 ± 29.48 ppm. These values are still within the broad range as reported in previous studies as shown in Table 1. According to Salleh et al. [16], the differences of the antioxidant activity in the propolis extract might be influenced by nearby botanical sources, geographical origin and types of solvent used in the extraction process. Nevertheless, the findings of this study closely resemble the values of IC_50_ (452.52–1027.29 ppm) for Indonesian propolis as reported by Fikri et al. [15].

In this study, the highest antioxidant activity was found in Gowa propolis (332.07 ± 6.12 ppm) followed by Lumajang (367.93 ± 24.60 ppm), and Bogor (396.47 ± 35.59 ppm). This agrees with their relatively high total phenolic content (93.46 ± 10.43–98.03 ± 13.94) as compared to propolis from other locations. The presence of phenolic and triterpenoids compounds (Table 2) contributes to the antioxidant activity of the propolis extract. From the results of principal component analysis as shown in Fig. 4, Gowa propolis, which had the highest antioxidant activity, was the only propolis in cluster 1. Although Lumajang and Bogor propolis had antioxidant activities that are not significantly different from Gowa, they were not in the same cluster. As such might indicate that the chemical compounds that influence the antioxidant activity are very specific and vary for each propolis sample as reflected in Table 2. The fact that the three propolis samples were not in the same cluster indicates that they might have different phenolic and triterpenoids compounds that influence the antioxidant activity.

**Fig. 9:**
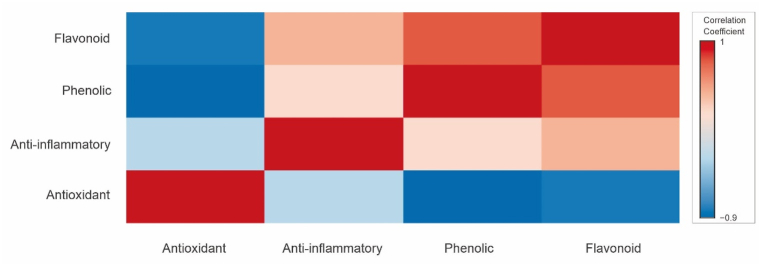
Correlation heatmap for antioxidant activity, anti-inflammatory activity, total phenolic content, and total flavonoid content of propolis extract from different geographical origin.

The values of IC_50_ were correlated with the total flavonoid and phenolic content of the propolis extract from various locations and the results are shown in Fig. 9. The results indicate that there was a significant negative correlation between the total flavonoid and phenolic content of the propolis extract with the values of IC_50_ of the DPPH. The coefficient correlation between total phenolic content and antioxidant activity was determined as −0.913 (P-value <0.01) whereas the coefficient correlation between total flavonoid content and antioxidant activity of was determined as −0.835 (P-value <0.01). The results resemble the previous findings by de Francisco et al., that there was a strong negative correlation between total flavonoid and phenolic content of propolis with the antioxidant activity [22].

### 3.6 Anti-inflammatory activity of propolis extract

**Fig. 10:**
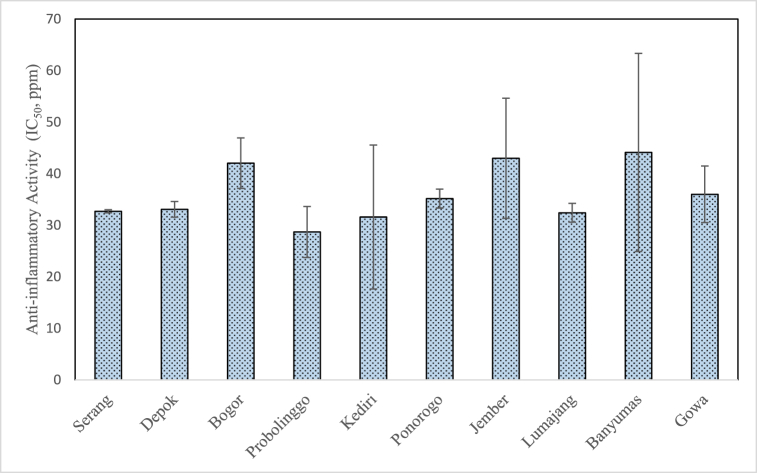
Anti-inflammatory activity of propolis extract from different geographical origin.

Fig. 10 shows the anti-inflammatory activity of propolis extract obtained in this study which lies in the range of 28.69 ± 4.95 to 44.12 ± 19.22 ppm. The anti-inflammatory activity of propolis extract obtained in this study was investigated based on the protein denaturation inhibition value where a low value of IC_50_ indicates that the concentration of the extract to inhibit protein denaturation is less and consequently the anti-inflammatory activity is considered as high. Studies that report in-vitro anti-inflammatory activity of propolis extract are very scarce but when compared to other studies, the values of IC_50_ determined in this study resembles the IC_50_ of *C. micranthum* extract (23.94–46.35 ppm) and has a greater anti-inflammatory activity as compared to the positive control particularly diclofenac potassium (267.82 ppm) [34].

From Fig. 9, it can be observed that the anti-inflammatory activity was not strongly correlated with the total flavonoid and phenolic content (coefficient correlation of 0.359 and 0.119, respectively). Nevertheless, the anti-inflammatory activity is influenced by the presence of triterpenoid compounds in the propolis extract. In this study, the highest anti-inflammatory activity was found in Probolinggo propolis with an IC_50_ value of 28.69 ± 4.95 ppm which may be due to the highest triterpenoid content particularly α-amyrin (8.88 %), β-amyrin (6.25 %) and lupeol (2.3 %) as shown in Table 2. As mentioned in the previous section, α, β-amyrin have anti-inflammatory activity and have the potential to combat acute pancreatitis. According to Liu et al., lupeol is a natural triterpenoid and a lot of studies have demonstrated that lupeol possesses a great anti-inflammatory activity [26]. As such may contribute to the greater anti-inflammatory activity of Probolingo propolis as compared to the other samples.

## 4 Conclusions

In brief, the total extract yield as well as their total flavonoid and phenolic content and in-vitro antioxidant and anti-inflammatory activity from 10 different locations in Indonesia had been investigated. The yield, bioactive compounds, and bioactivity of the propolis extract varies from one geographical origin to another. The yield of propolis extract investigated in this study lies in the range of 26.25 ± 1.76 to 43.25 ± 3.60 % whereas the total flavonoid and phenolic content of the propolis extract varies from 0.70 ± 0.08–57.76 ± 0.67 mg QE/g and 50.03 ± 3.40–98.03 ± 13.94 mg GAE/g, respectively. The findings of this study discovered that the antioxidant activity of the propolis extract increased significantly with higher amounts of total phenolic and flavonoid content. This study also detected 36 volatile compounds present in the propolis extract. The results of principal component analysis highlight that propolis extract obtained from different islands belong to different cluster of chemical compounds with triterpenoid as the major substances. The presence of anti-inflammatory compounds particularly α, β-amyrin and lupeol in the propolis extract highlights the potential of propolis in health and medicine application.

## Data availability statement

Data will be made available upon request.

## CRediT authorship contribution statement

**Muhammad Yusuf Abduh:** Writing – review & editing, Writing – original draft, Validation, Supervision, Resources, Project administration, Funding acquisition, Formal analysis, Conceptualization. **Tri Ramadianti Shafitri:** Writing – original draft, Software, Investigation, Formal analysis, Data curation. **Elfahmi Elfahmi:** Writing – review & editing, Funding acquisition, Conceptualization.

## Declaration of competing interest

The authors declare that they have no known competing financial interests or personal relationships that could have appeared to influence the work reported in this paper.
